# Resistant *Escherichia coli* isolated from wild mammals from two rescue and rehabilitation centers in Costa Rica: characterization and public health relevance

**DOI:** 10.1038/s41598-024-57812-6

**Published:** 2024-04-05

**Authors:** Rita Fernandes, Raquel Abreu, Isa Serrano, Roger Such, Encarnación Garcia-Vila, Sandy Quirós, Eva Cunha, Luís Tavares, Manuela Oliveira

**Affiliations:** 1 CIISA - Centro de Investigação Interdisciplinar Em Sanidade Animal, Faculdade de Medicina Veterinária, Universidade de Lisboa, Av. da Universidade Técnica, 1300-477 Lisbon, Portugal; 2 AL4AnimalS - Associate Laboratory for Animal and Veterinary Sciences, Lisbon, Portugal; 3 Jaguar Rescue Center, Limón, Costa Rica; 4 Alturas Wildlife Sanctuary, Puntarenas, Costa Rica; 5 cE3c - Centre for Ecology, Evolution and Environmental Changes & CHANGE - Global Change and Sustainability Institute, Faculdade de Ciências, Universidade de Lisboa, Lisbon, Portugal

**Keywords:** Antimicrobial resistance, Public health, Rehabilitation, Virulence factors, Wildlife, Antimicrobials, Bacteria, Bacteriology

## Abstract

This study aimed to characterize the antimicrobial resistance (AMR) and virulence profiles of 67 *Escherichia coli* isolates obtained from faecal samples of 77 wild mammals from 19 different species, admitted in two rescue and rehabilitation centers in Costa Rica. It was possible to classify 48% (n = 32) of the isolates as multidrug-resistant, and while the highest resistance levels were found towards commonly prescribed antimicrobials, resistance to fluoroquinolones and third generation cephalosporins were also observed. Isolates obtained from samples of rehabilitated animals or animals treated with antibiotics were found to have significantly higher AMR levels, with the former also having a significant association with a multidrug-resistance profile. Additionally, the isolates displayed the capacity to produce α-haemolysins (n = 64, 96%), biofilms (n = 51, 76%) and protease (n = 21, 31%). Our results showed that AMR might be a widespread phenomenon within Costa Rican wildlife and that both free-ranging and rehabilitated wild mammals are potential carriers of bacteria with important resistance and virulence profiles. These results highlight the need to study potential sources of resistance determinants to wildlife, and to determine if wild animals can disseminate resistant bacteria in the environment, potentially posing a significant threat to public health and hindering the implementation of a “One Health” approach.

## Introduction

Antimicrobial resistance (AMR) has emerged as one of the leading challenges of the twenty-first century, threatening public health, food security and economic development at a global scale^1–3^. Antimicrobial drugs have rapidly become less effective, and even ineffective, quickly outpacing the available treatment alternatives^4,5^.

The driving forces of AMR are known to be multisectoral, resulting from complex interactions within the human-animal-environment interface. Therefore, it is currently agreed upon that this global problem must be approached within an “One Health” framework that implements collaborative and interdisciplinary strategies^6–8^. However, this approach can only be successful once we understand the role of each sector and how their interactions might contribute to the emergence and dissemination of antimicrobial resistance^2,3,7^.

Despite the large and growing literature on AMR, particularly in clinical settings, studies on the role of the environmental compartment in this global problem are still lacking, particularly focusing on the role of wild species^9–12^.

Over the last few decades, clinically relevant antimicrobial-resistant bacteria have been identified in numerous wildlife species across all ecosystems, including from remote regions such as Antarctica^13–16^. AMR in wildlife is particularly interesting since, theoretically, free-living wild animals are not intentionally treated with antimicrobials, and therefore direct exposure to these compounds should be rare. This indicates that the resistant strains and genes found in wild species probably emerged as a consequence of the interaction between these animals and the humans, livestock and domestic animals which share the same environment^9,12,17^. A growing human population and the increasing fragmentation and urbanization of natural habitats have forced this interaction to happen more frequently and on a greater scale^4,11^. Although AMR in wildlife appears to be a function of the level of anthropization of the occupied habitat, sources, directionality, and specific patterns within this interaction are yet to be definitively assigned^15,16^.

Wild animals have been established as useful sentinels, since resistances on their microbiome appear to mirror the ones present in their environment. However, they have also been recognized as possible reservoirs, vectors and secondary sources of AMR bacteria for humans and domestic animals^12,14,18^. One of the main concerns is their potential to not only harbour important resistance and virulence genes, but also serve as melting pots for novel gene combinations with increased pathogenic potential^12,14,19^.

It is then increasingly important to understand the role of wildlife in the dynamics of AMR, as this knowledge gap might hinder the implementation of a successful holistic strategy. However, the methods applied to study the evolution and transmission of AMR in clinical settings are challenging to apply to wild ecosystems^11^. Therefore, wildlife rescue and rehabilitation facilities can provide a unique opportunity to study AMR in wild species, as they allow the collection of samples from wild animals without interfering directly with the natural habitats^20,21^.

This study aimed to characterize the antimicrobial resistance and virulence profiles of *Escherichia coli* isolates obtained from faecal samples of wild mammals admitted in two rescue and rehabilitation centers in Costa Rica. Furthermore, it also aimed to assess if human care could contribute to significant differences in the resistance and pathogenic profiles of wild isolates. Other factors were also analysed to check for potential associations with these profiles, such as cause of admission, implementation of antibiotic therapy and time in rehabilitation.

This study aims to contribute for the assessment of the role of wild mammal species as potential carriers and spreaders of important resistance and virulence profiles for human and veterinary medicine.

## Materials and methods

### Sampling location

The samples analyzed in this study were collected in two distinct rescue and rehabilitation centers in Costa Rica, for a total period of 18 weeks.

**Figure 1:**
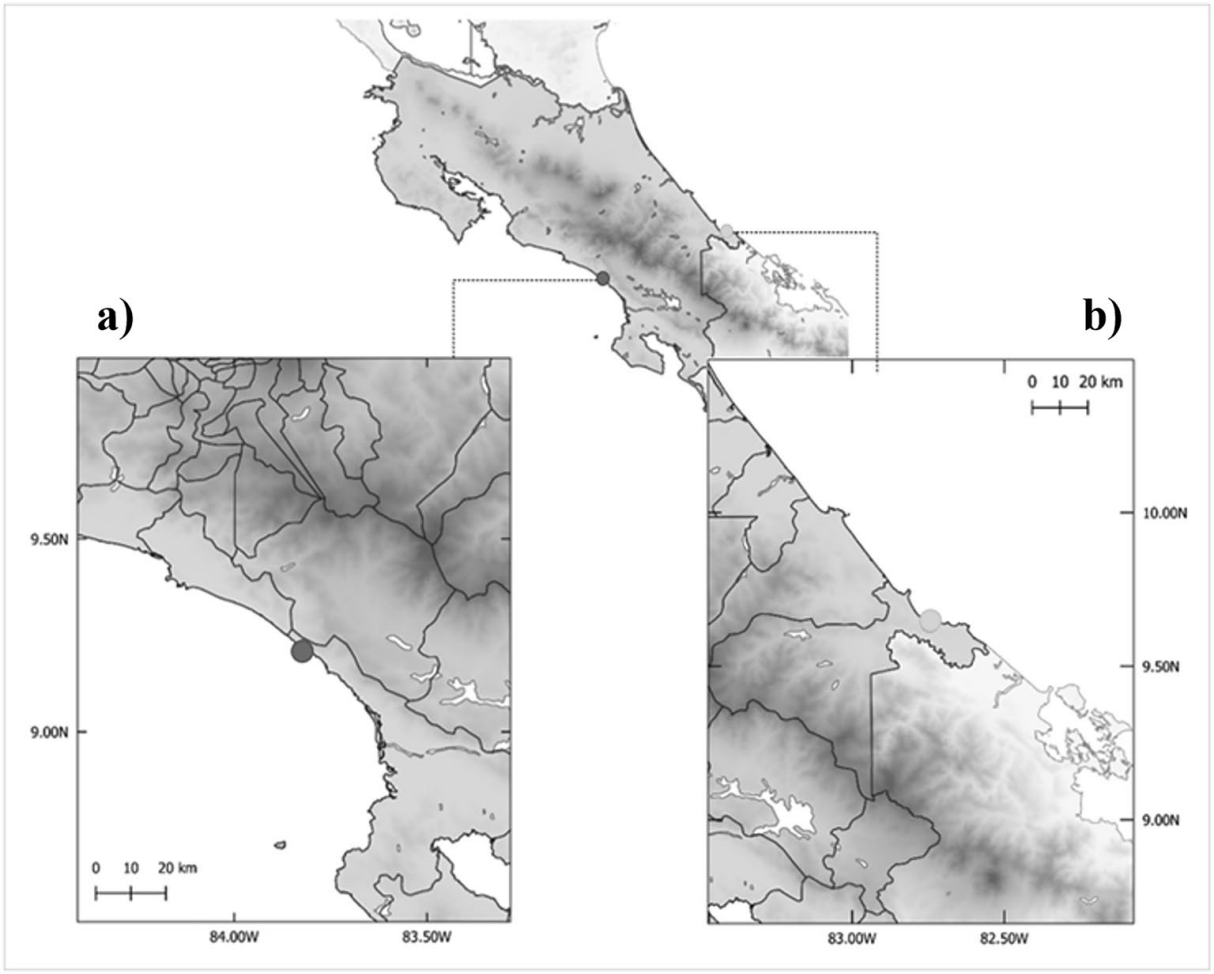
Location of the rescue and rehabilitation centers where the sampling took place. (**a**)—Alturas Wildlife Sanctuary, Dominical, Puntarenas, signalled with a dark green circle. (**b**)—Jaguar Rescue Center, Punta Cocles, Limón, signalled with a light green circle. (Original map created in QGIS, version 3.28.1). Both rescue centers are located within forestry areas and near prestigious national parks, having a rich fauna and flora biodiversity. However, they are also located near busy areas with a high tourism development, such as Uvita, Dominical and Puerto Viejo de Talamanca.

From December 2021 to February 2022, samples were collected at Alturas Wildlife Sanctuary, Dominical, Puntarenas (Lat: 9° 13′ 13.1484′′ N; Long: 83° 49′ 23.7324′′ W), located in the Southern Pacific area (Fig. 1a). From March 2022 to May 2022, samples were collected at Jaguar Rescue Center, Punta Cocles, Limón (Lat: 9° 38′ 30.1308′′ N; Long: 82° 43′ 23.1852′′ W), located in the Southern Caribbean Coast (Fig. 1b).

### Sampled animals

The present study was approved by MINAE (Ministerio Del Ambiente y Energía) and SINAC (Sistema Nacional de Áreas de Conservación), being performed in accordance with the research permit R-SINAC-PNI-ACLAC-026-2021. The author also had a scientific collection license (ACLAC-130-2021). This study did not involve animal experiments or collection of specimens. None of the procedures implicated the disturbance of wild animals in their natural habitat and the handling time for sample collection did not exceed 2 min, as was performed by trained veterinarians.

Only wild mammal species were selected for this study, with sampling being made in one of two distinct occasions: (1) when the animals were first admitted to the rescue center and before the implementation of any treatment—referred to as “admitted animals”; or (2) when the animals were doing the last check up before being released back to the wild or moved to a pre-release enclosure—referred to as the “rehabilitated animals”. Therefore, samples were collected from two groups of animals with very different status regarding their direct contact with human care.

The cause of admission was registered for every sampled animal, using a total of eight categories: “Injury”, “Sickness”, “Orphan”, “Electrocution”, “Traffic Accident”, “Translocation”, “Confiscation” and “Death”. An animal was classified as “Injured” when the cause of admission was mostly of a physical nature, such as fractured limbs, wounds or falling from trees. On the other hand, animals that presented weakness, poor body condition or visible signs of disease, were classified as “Sickness”. “Translocation” included the animals rescued from non-ideal locations, such as a person’s house or a high traffic area. These animals were usually brought to the clinic only for a check-up and were released on the same day in an appropriate location. An animal was labeled as “Confiscated” when it was illegally being kept by someone and the local authorities were involved.

The animals were divided by age into “Baby”, “Juvenile” and “Adult”. The distinction between a baby and a juvenile was mostly done according to size and level of independence, always considering the species’ characteristics.

The total rehabilitation time was divided in three intervals: less than 6 months, between 6 months and 1 year, and more than 1 year.

Every mammal that was admitted or released from the rescue centers during the 18 weeks-essay was sampled, unless they were too young, small, injured or stressed, as in these cases the sampling procedure could interfere negatively with their wellbeing.

### Sampling technique

AMIES swabs (VWR, Leuven, Belgium) were used to collect rectal samples from the selected animals. The swabs were inserted approximately 2 cm into the rectum, and rotative movements were used to collect faecal material from the rectal dilation^22,23^. All animals were adequately restrained, with handling time not exceeding 2 min per animal for the collection. The stress was minimized by providing a calm environment and using gentle and slow movements. When possible, the sampling was done under sedation (if that was already part of the animal’s treatment or diagnostic plan). The samples were kept at 4 °C until transportation to the Microbiology and Immunology Laboratory from the Faculty of Veterinary Medicine of the University of Lisbon for further processing.

The following information was collected regarding each sample: date of sampling, place of sampling (Alturas Wildlife Sanctuary or Jaguar Rescue Center), animal sampled (“admitted” or “rehabilitated”), identification of the animal (species, sex and age), date of admission, cause of admission (e.g. electrocuted, orphaned, injured), previous antimicrobial treatment and, when applied, date of release from the rescue center and total time spent in rehabilitation.

### Isolation and Identification of Escherichia coli

The collected samples were inoculated directly on MacConkey (MK) agar medium (VWR, Leuven, Belgium) and incubated at 37 °C for 24 h^24,25^. After incubation, the macroscopic morphology of the developed colonies was assessed to select for bright pink (lactose fermenting) colonies surrounded by a pink precipitate halo. A minimum of 4 colonies per culture were then isolated in new MacConkey agar plates and further incubated at 37 °C for 24 h, to ensure the culture’s purity. From these, one cultural type was randomly selected for identification, after inoculation in Brain Heart Infusion (BHI) agar (VWR, Leuven, Belgium), and incubation at 37 °C for 24 h. Whenever the four isolated colonies presented different macroscopic characteristics (e.g., differences in the pink shade or mucoid texture), one isolate from each culture type was selected for identification.

An adaptation of IMViC test (Indole, Methyl Red, Voges-Proskauer, Citrate) was used for presumptive *Escherichia coli* identification^26,27^, comprising of three different culture media: Simmons Citrate Agar (Oxoid, Hempshire, UK), Sulphide Indole Motility Agar (Merck, Darmstadt, Germany) and Voges-Proskauer Medium (Oxoid, Hempshire, UK). After inoculation and incubation at 37 °C for 24 h, an isolate would be identified as *E. coli* if it presented the following characteristics: negative for citrate usage, positive for indole production, negative for hydrogen-sulphide production, motile and VP negative^23,26,27^.

### Evaluation of the isolates’ antimicrobial resistance profile

The antimicrobial susceptibility profile of the *E. coli* isolates under study were evaluated using a standardized disk diffusion method, following the guidelines of the Clinical and Laboratory Standards Institute (CLSI)^28,29^.

A total of 15 antibiotics (Oxoid, Hampshire, UK) from 10 different classes were tested: aminoglycosides—gentamicin (CN,10 µg), amikacin (AK, 30 µg); penicillins—ampicillin (AMP, 10 µg); first and third generation cephalosporins—cephalexin (CL, 30 µg), ceftazidime (CAZ, 30 µg), ceftiofur (EFT, 30 µg); fluoroquinolones—ciprofloxacin (CIP, 5 µg), enrofloxacin (ENR, 5 µg); carbapenems—imipenem (IMP, 10 µg), meropenem (MEM, 10 µg); tetracyclines—oxytetracycline (OT, 30 µg); beta-lactam with beta-lactamase inhibitor—amoxicillin/clavulanic acid (AMC, 30 µg); phenicols—chloramphenicol (C, 30 µg); macrolides—azithromycin (AMZ, 15 µg); and sulfonamides—trimethoprim/sulfamethoxazole (SXT, 25 µg).

These compounds were selected based on their use in human and veterinary medicine, including the specific cases of the two wildlife rehabilitation facilities where the sampling took place. The reference strain *Escherichia coli* ATCC® 25922 was used for quality control.

After incubation, the inhibition zones of each compound were measured, allowing to classify the isolates as susceptible (S), intermediate (I) and resistant (R), according to the breakpoints established by the CLSI guidelines^28,29^, apart from cephalexin (CL, 30 µg), for which the EUCAST breakpoints were considered^30^. A 10% replica was performed to check for reproducibility, by repeating the susceptibility tests of randomly selected isolates.

### Evaluation of the isolates’ virulence profile

The phenotypic virulence profile of the isolates was assessed through their ability to produce compounds associated with bacterial pathogenicity. A total of 6 virulence factors were evaluated: protease, DNAse, gelatinase, lecithinase, haemolysin and biofilm production. The isolates were cultured in BHI agar (VWR, Leuven, Belgium) at 37 °C for 24 h before being inoculated in different specific media. A 10% replica was performed for each test to check for reproducibility.

Protease expression was assessed using Skim Milk agar plates, constituted by Skim Milk powder (VWR, Leuven, Belgium) supplemented with bacteriological agar (VWR, Leuven, Belgium), incubated at 37 °C for 48 h. *Pseudomonas aeruginosa* ATCC 27853™ was used as the positive control and *Staphylococcus aureus* ATCC® 29213 as the negative. The isolates were classified as positive for protease production when a halo of transparency or reduced opacity was formed around the bacterial growth^23,27,31^.

Deoxyribonuclease (DNAse) activity was evaluated by inoculation in DNAse Agar (Remel, California, USA) plates supplemented with 0.01% Toluidine Blue (Merck, Darmstadt, Germany), followed by incubation at 37 °C for 48 h. *S. aureus* ATCC 25923 and *E. coli* ATCC 25922 were used as positive and negative controls, respectively. A positive isolate would appear with a pink or purple reaction, or a clear halo surrounding the bacterial growth^27,32,33^.

Gelatinase production was detected using test tubes containing Nutrient Gelatin medium (Oxoid, Hampshire, UK). The tubes were incubated at 37 °C for 48 h, after which they were incubated at 4 °C for 30 min. A positive gelatinase activity would be presented as a partial or total liquefaction of the medium. The positive and negative controls used were, respectively, *P. aeruginosa* ATCC Z25.1 and *E. coli* ATCC 25922^27,33^.

Lecithinase activity was determined using Tryptic Soy Agar (VWR, Leuven, Belgium) plates supplemented with 10% egg yolk emulsion (VWR, Leuven, Belgium). After incubation at 37 °C for 48 h, positive isolates were identified by the appearance of a white, diffuse, and opaque precipitation around the bacterial growth. *P. aeruginosa* ATCC 27853 was tested as the positive control, while *E. coli* ATCC 25922 was tested as the negative^23,27,33^.

Haemolysin production was evaluated using Columbia Agar supplemented with 5% sheep blood (bioMérieux SA, Marcy l’Étoile, France), incubated at 37 °C for 48 h, with confirmation of the results at 72 h. If a complete clearing halo appeared around the bacterial colony, the isolate was considered β-haemolytic. If the haemolysis was not complete, resulting in a yellow/greenish discoloration around the growth, it was considered α-haemolytic. If no change appeared around the colony, the isolate was considered Ɣ-haemolytic or non-haemolytic^23,34,35^.

Lastly, biofilm production was assessed using Congo Red Agar plates, composed of 3.7% Brain Heart Infusion broth (VWR, Leuven, Belgium), 5% Sucrose (Sigma, Steinheim, Germany), 1.4% Bacteriological Agar (VWR, Leuven, Belgium) and 0.08% Congo Red reagent (Sigma, Steinheim, Germany). The plates were incubated at 37 °C for 72 h, with results read at 24, 48 and 72 h. Black colonies, either fully or partially, with crystalline and dry consistency were considered positive for biofilm formation. *P. aeruginosa* ATCC 27853 and *E. coli* ATCC 25922 were respectively used as positive and negative controls^34–36^.

### Statistical analysis

Statistical analysis was performed using R (R Foundation for Statistical Computing, Vienna, Austria, version 4.2.2), RStudio (Posit Software, Boston, USA, version 2022.07.2 + 575) and Microsoft Office Excel 365 (Microsoft Corporation, Redmond, USA).

The Multiple Antimicrobial Resistance (MAR) Index and the Virulence Index (V. Index) values were determined for all *E. coli* isolates, according to the following equations: MAR Index = (number of antimicrobials for which isolates tested resistant / number of antimicrobials tested); V. Index = (number of positive virulence factors / number of virulence factors tested)^23^. As the MAR and Virulence Indexes did not follow a normal distribution, non-parametric tests were applied to evaluate these variables and possible associations with other factors.

According to the definitions established by Magiorakos et al.^37^ for *Enterobacteriaceae,* the isolates were further classified into Multidrug-Resistant (MDR) when they were non-susceptible to at least one antimicrobial from 3 or more antimicrobial categories.

The Mann–Whitney U test was used to find possible differences in these indexes according to the sampling location, sex of the animals, animal status (“admitted” versus “rehabilitated”), administration of antibiotic therapy, and isolates’ biofilm production ability. Similarly, the Kruskal–Wallis test was applied to test possible differences in the index values according to the animals’ species, age, feeding behavior, cause of admission and time in rehabilitation.

To evaluate the relationships between MDR and sampling location, antibiotic therapy and isolates’ biofilm-forming ability, a Chi-square test was performed. A Fisher’s exact test was used to analyse possible associations between biofilm production and the different virulence factors, as well as between *E. coli* isolation and the feeding habits of the sampled animals. The correlation between MAR and Virulence Indexes was analysed with the Spearman correlation test.

Significant differences were calculated at 0.05 (two-tailed) levels of significance.

## Results

### Sampled animals

A total of 77 animals were sampled (Table S1), 38% (n = 29) in Alturas Wildlife Sanctuary and 62% (n = 48) in Jaguar Rescue Center. Out of the total animals under study, 71% (n = 55) were sampled upon admission (“Admitted Animals”), and 29% (n = 22) were sampled before release (“Rehabilitated Animals”). A total of 19 different animal species were sampled, with three of them having a considerable higher number of sampled individuals: *Choloepus hoffmanni* (Two-Toed Sloth, n = 17), *Alouatta palliata* (Howler Monkey, n = 11) and *Bradypus variegatus* (Three-Toed Sloth, n = 9). The remaining species can be consulted in Table 1.

**Table 1:** Number of samples (n = 77) and *Escherichia coli* isolates (n = 67) obtained from each animal species.

Scientific Name	Common Name	Nr. Isolates	Nr. Samples
*Choloepus hoffmanni*	Hoffmann's Two-Toed Sloth	16	17
*Alouatta palliata*	Mantled Howler Monkey	11	11
*Bradypus variegatus*	Brown-Throated Three-Toed Sloth	7	9
*Cuniculus paca*	Paca	5	5
*Dasyprocta punctata*	Central American Agouti	4	6
*Tamandua mexicana*	Northern Tamandua	4	5
*Didelphis marsupialis*	Common Opossum	3	4
*Coendou mexicanus*	Mexican Hairy Dwarf Porcupine	2	1
*Eira barbara*	Tayra	2	1
*Leopardus pardalis*	Ocelot	2	2
*Philander opossum*	Common Gray Four-Eyed Opossum	2	2
*Procyon cancrivorus*	Crab-Eating Racoon	2	2
*Procyon lotor*	Northern Raccoon	2	3
*Cebus imitator*	White-Faced Capuchin Monkey	2	1
*Caluromys derbianus*	Central American Woolly Opossum	1	3
*Leopardus wiedii*	Margay	1	2
*Herpailurus yagouaroundii*	Jaguarundi	1	1
*Saimiri oerstedii*	Central American Squirrel Monkey	0	1
*Artibeus obscurus*	Dark Fruit-Eating Bat	0	1

**Figure 2:**
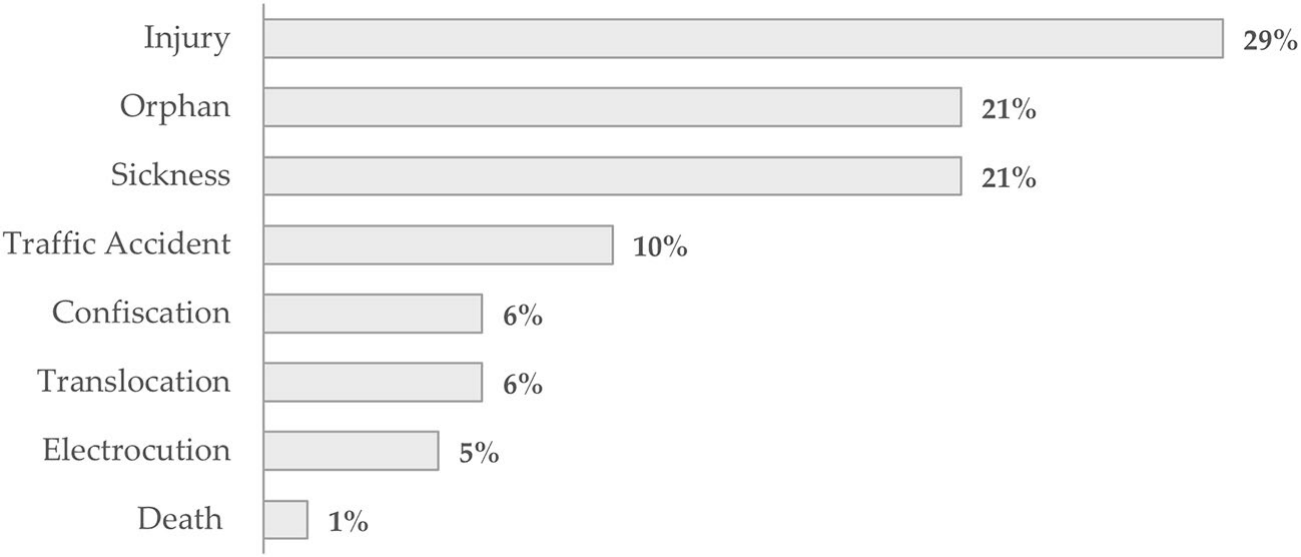
Causes of admission of the sampled animals (n = 77).

The three main causes of admission were “Injury” (29%, n = 22), “Orphan” (21%, n = 16) and “Sickness” (21%, n = 16). The remaining five causes can be consulted in Fig. 2.

The average rehabilitation time in Alturas Wildlife Sanctuary was 70 days and in Jaguar Rescue Center was 411 days. Overall, the total rehabilitation periods ranged from 24 h to 1125 days.

### Identification of the isolates

From the 77 faecal samples collected, it was possible to obtain a total of 105 isolates with a macroscopic profile in MacConkey agar (MK) compatible with lactose fermenting *Enterobacteriaceae*, which were further identified using the IMViC test. From these, it was possible to identify 67 isolates as *Escherichia coli*, obtained from 57 different animals of 17 different animal species. The number of isolates obtained per animal species can be consulted in Table 1.

Out of the *E. coli* isolates obtained, 47 (70%) were isolated from samples of “admitted” animals, while the remaining 20 (30%) were obtained from samples of “rehabilitated” individuals.

The majority of isolates were obtained from samples of herbivores (67%, n = 45) and omnivores (21%, n = 14), with a much lower percentage being isolated from carnivores (6%, n = 4) and myrmecovores (6%, n = 4). No significant statistical association was found between the odds of obtaining a positive *E. coli* isolate and the feeding habits of the sampled animals (p > 0.05), even though it was possible to identify *E. coli* isolates in 72% (n = 45) of the bacterial cultures from herbivore mammals samples, but only in 57% (n = 4) of the cultures from carnivore animals samples and 48% (n = 14) from omnivores samples.

### Characterization of the isolates’ antimicrobial resistance profile

According to the definitions established by Magiorakos et al.^37^, 48% (n = 32) of the studied isolates can be classified as multidrug-resistant (MDR), since they were non-susceptible to at least one antibiotic from three or more antimicrobial categories.

It was possible to observe that 93% of the isolates (n = 62) were resistant or intermediately resistant to at least one of the fifteen compounds analysed, and that isolates resistant to at least one of the antimicrobials tested were found in samples from all the 17 animal species analysed. The highest levels of resistance were shown towards cephalexin (58%, n = 39), followed by ampicillin (43%, n = 29) and oxytetracycline (22%, n = 15). All isolates were susceptible to the aminoglycosides and carbapenems under study (Table 2).

**Table 2:** Antimicrobial susceptibility of the *E. coli* isolates under study (n = 67).

Antimicrobial class	Antimicrobial compound (dose)	*E. coli* Isolates [n (%)]^a^		
		S^b^	I^c^	R^d^
Aminoglycosides	Amikacin (30 µg)	67 (100)	0 (0)	0 (0)
	Gentamicin (10 µg)	67 (100)	0 (0)	0 (0)
β-lactam with β-lactamase inhibitor	Amoxicillin/Clavulanic Acid (30 µg)	58 (87)	8 (12)	1 (1)
Carbapenems	Imipenem (10 µg)	67 (100)	0 (0)	0 (0)
	Meropenem (10 µg)	67 (100)	0 (0)	0 (0)
Cephalosporins	Cephalexin (30 µg)	28 (42)	0 (0)	39 (58)
	Ceftazidime (30 µg)	61 (91)	1 (1)	5 (7)
	Ceftiofur (30 µg)	64 (96)	0 (0)	3 (4)
Fluoroquinolones	Ciprofloxacin (5 µg)	60 (90)	2 (3)	5 (7)
	Enrofloxacin (5 µg)	60 (90)	1 (1)	6 (9)
Macrolides	Azithromycin (15 µg)	61 (91)	0 (0)	6 (9)
Penicillins	Ampicillin (10 µg)	19 (24)	22 (33)	29 (43)
Phenicols	Chloramphenicol (30 µg)	64 (96)	0 (0)	3 (4)
Sulphonamides	Trimethoprim/Sulfamethoxazole (25 µg)	66 (99)	0 (0)	1 (1)
Tetracyclines	Oxytetracycline (30 µg)	28 (42)	24 (36)	15 (22)

^a^n (%), number and percentage of isolates; ^b^S, Susceptible; ^c^I, Intermediate; ^d^R, Resistant.

A total of 12 isolates were obtained from samples of animals that received at least one of the following antibiotic treatments: amoxicillin with clavulanic acid, enrofloxacin, trimethoprim-sulfamethoxazole or metronidazole. The average duration of the antimicrobial therapy upon the moment of sampling ranged from 12 h to 15 days, with an average of 7 days.

The MAR Index mean value of the isolates was 0.112, with the highest value observed being 0.6.

**Figure 3:**
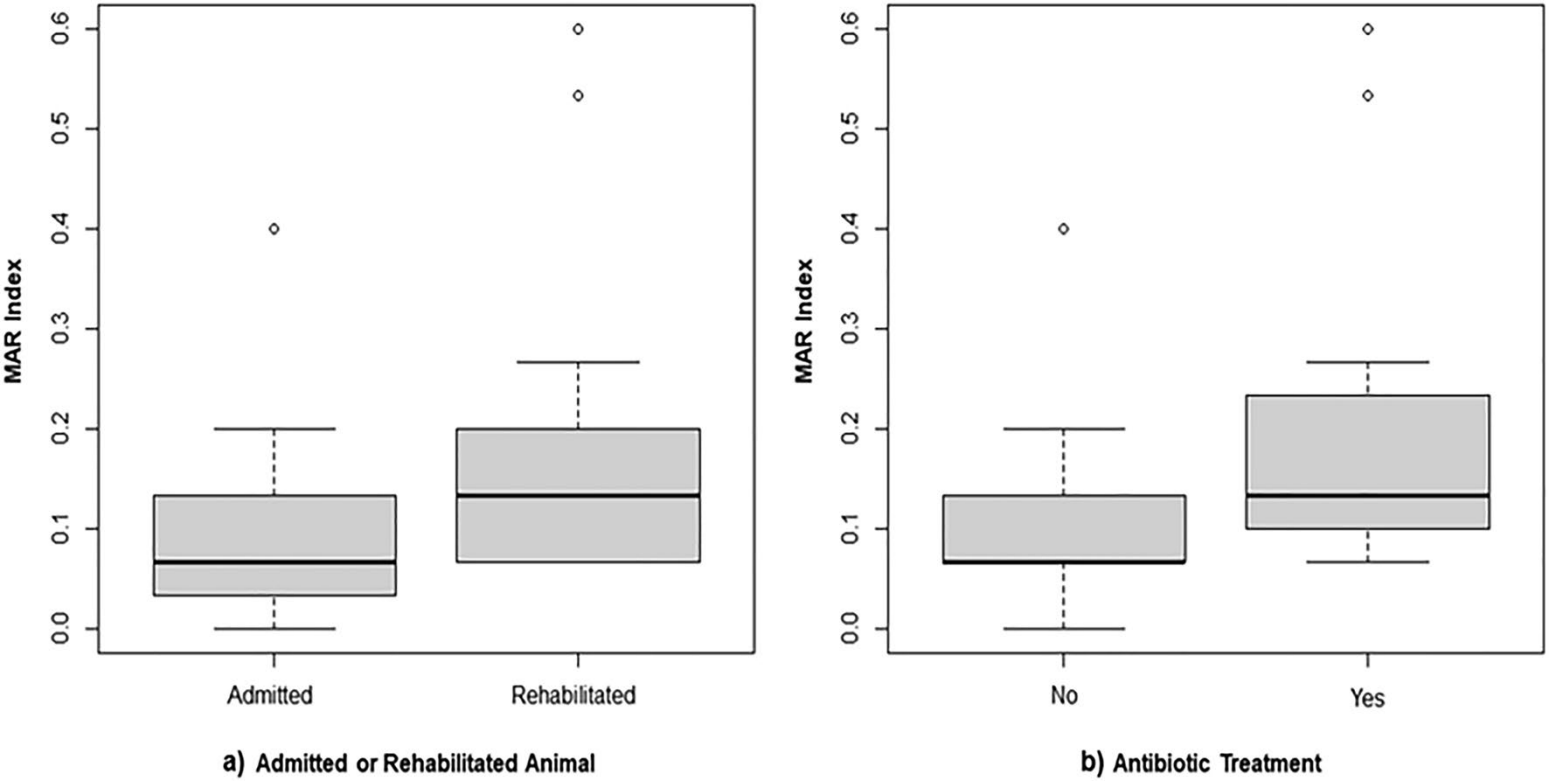
Boxplot representing the MAR Index according to (**a**) the status of the sampled animal (admitted or rehabilitated), and (**b**) the implementation of antibiotic treatment (yes or no) (n = 67).

Significant statistical differences (p = 0.01) were found between the MAR Index values of isolates obtained from animals that received antibiotic treatment (MAR Index = 0.206) and from the ones that did not (MAR Index = 0.092) (Fig. 3a).

Significant statistical differences (p = 0.0008) were also found between the MAR Index values of isolates obtained from “admitted” and “rehabilitated” animals, with rehabilitated animals having a higher MAR Index mean value (MAR Index = 0.180) when compared to the admitted ones (MAR Index = 0.083) (Fig. 3b).

Additionally, a statistically significant association (p = 0.03) was found between the multidrug-resistance profile of the isolates and the status of the sampled animals (“admitted” or “rehabilitated”), with isolates from rehabilitated animals presenting a higher proportion of multidrug-resistance (70%, n = 14) when compared to the ones obtained from admitted animals (40%, n = 19).

**Figure 4:**
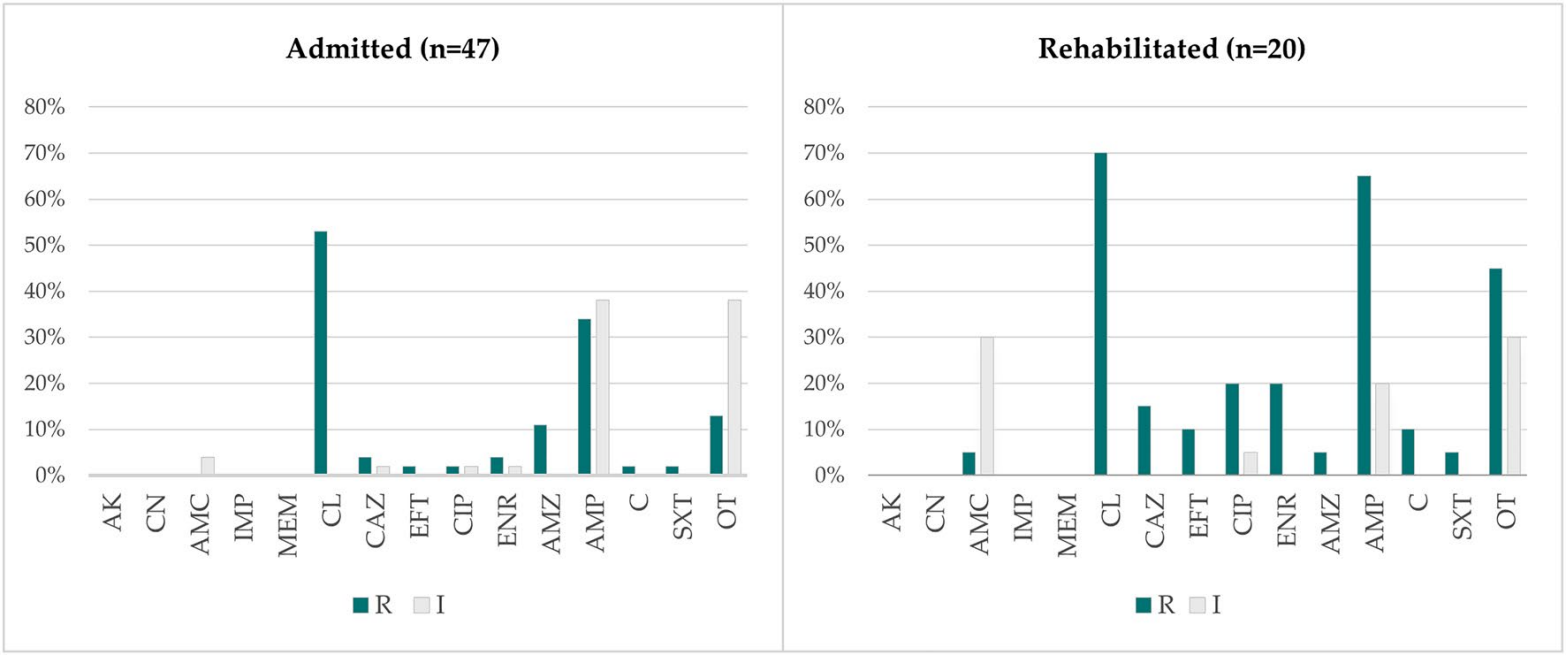
Comparison of the non-susceptibility levels of the *E. coli* isolates obtained from samples of rehabilitated and admitted animals (n = 67). R, Resistant, I, Intermediate; AK, Amikacin, CN, Gentamicin, AMC, Amoxicillin/Clavulanic Acid, IMP, Imipenem, MEM, Meropenem, CL, Cephalexin, CAZ, Ceftazidime, EFT, Ceftiofur, CIP, Ciprofloxacin, ENR, Enrofloxacin, AZM, Azithromycin, AMP, Ampicillin, C, Chloramphenicol, SXT, Trimethoprim/Sulfamethoxazole, OT, Oxytetracycline.

It was also possible to observe that the isolates obtained from rehabilitated animals, apart from presenting generally higher resistance levels, also had a higher percentage of resistance to critically important antimicrobials, such as third generation cephalosporins (ceftazidime and ceftiofur) and fluoroquinolones (enrofloxacin and ciprofloxacin) (Fig. 4).

**Figure 5:**
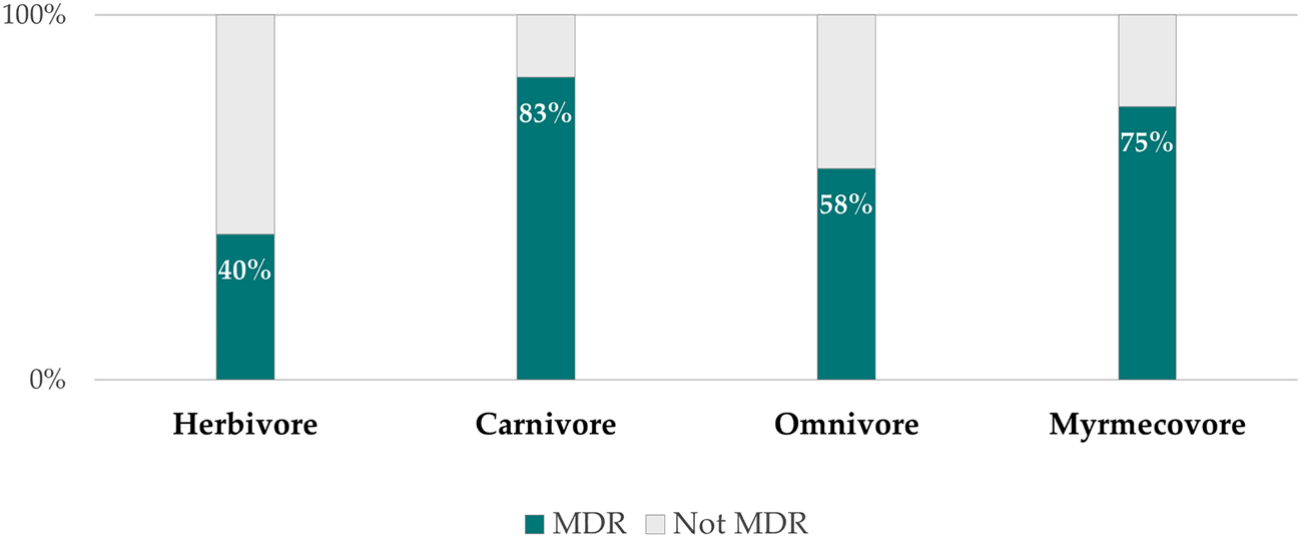
Comparison of the proportion of multidrug-resistant isolates, according to the feeding habits of the sampled animal of origin (n = 67). MDR, Multidrug-resistant.

No statistically significant differences (p > 0.05) were found between the MAR Index values according to the sex, age or species of the sampled animals, cause of admission, time in rehabilitation or location of sampling. Additionally, no significant association was observed between the isolates’ ability to produce biofilms and both the MAR Index and multidrug resistance profile of the isolates (p > 0.05). There was also no significant association (p > 0.05) between the multidrug-resistance profile and the feeding habits of the sampled animals, even though there was a proportionately higher number of MDR isolates obtained from carnivores (83.3%, n = 5), when compared to the remaining groups, particularly herbivores (40%, n = 18) (Fig. 5).

### Characterization of the isolates’ virulence profile

Regarding the phenotypic expression of virulence factors, most of the isolates were able to produce α-haemolysin (n = 64, 96%) and biofilms (n = 51, 76%). Protease production was observed in 31% of the isolates (n = 21). A very low number of isolates demonstrated DNAse (n = 5, 7%) and gelatinase (n = 1, 1%) activities. None of the isolates were able to produce lecithinase.

The Virulence Index mean value of the isolates under study was 0.353, with the highest value presented being 0.833. Only one isolate was not able to produce any virulence factors.

No significant statistical differences (p > 0.05) were found between the V. Index of the isolates and the different factors evaluated: animals’ species, sex and age, cause of admission, location of sampling, admitted or rehabilitated animal, time in rehabilitation, antibiotic treatment and isolates’ biofilm production ability. There was also no association found between biofilm production and the remaining virulence factors tested (p > 0.05).

There was, however, a significant correlation found between the MAR and the Virulence Indexes (p = 0.03). The Spearman correlation coefficient was -0.2597, which corresponds to a weakly negative relationship. This means that when the MAR Index was higher, the Virulence Index tended to be lower and vice-versa.

## Discussion

The present study provided some relevant insights regarding the most common antimicrobial resistance profiles and virulence factors currently present in bacteria from Costa Rican wildlife, as well as the influence that wildlife rehabilitation might have on bacterial pathogenic potential.

The use of rectal swabs with Amies transport medium (VWR, Leuven, Belgium) constituted a quick, effective and non-traumatic sampling technique, as described by Fernandes et. al^23^. This method has been widely used for sample collection in wildlife, being considered reliable for Gram-negative bacteria isolation, while also allowing a long-distance transportation and delayed analysis^27,38^.

It was observed that the majority of isolates under study (93%) were non-susceptible to at least one of the fifteen compounds tested, and resistant isolates were found in samples from all 17 analysed animal species. This is a worrying result, since it suggests that antimicrobial resistance might be a widespread phenomenon across Costa Rican wildlife.

The highest levels of resistance were shown towards cephalexin (58%), followed by ampicillin (43%) and oxytetracycline (22%). These results are supported by multiple studies performed on wildlife across the globe, both in free-ranging and captive conditions, in which first generation cephalosporins, penicillins and tetracyclines are consistently responsible for the highest resistance profiles, although not always necessarily in this order^39–45^.

These results are also in accordance with the available data on AMR and antibiotic usage in Costa Rica. Penicillins and their derivatives are the most consumed antimicrobials in the country, followed by cephalosporins, carbapenems and tetracyclines^7,46^. Studies from Costa Rican healthcare facilities have consensually reported that *E. coli* isolates present the highest levels of resistance towards penicillins and first generation cephalosporins^47–49^, and concordant results were also found in healthy dogs from Costa Rican households^50^.

With penicillins, first generation cephalosporins and tetracyclines being widely used for decades in both human and veterinary healthcare, the presence of bacteria resistant to these compounds was expected to some degree in the sampled animals^4,51^. However, the isolates under study also demonstrated resistance to critically important antimicrobials with more restrictive applications, namely third-generation cephalosporins and fluoroquinolones.

While studies on fluoroquinolone resistance are still fairly limited in wild mammals, they have gained special focus among scavenger avian species, particularly vultures, as they commonly feed on carrion from livestock previously subjected to antibiotic therapy^52–54^. This could be relevant and interesting for future studies, as Costa Rica has a vast population of New World vultures^55^ that could be involved in the dissemination of fluoroquinolone resistance to wild species.

Resistance to the third-generation cephalosporins ceftazidime and ceftiofur was observed in respectively 7% and 5% of the isolates under study. Similar results in wild species have been reported in previous studies^23,41,44^, and while still being relatively low numbers, their detection raises concern due to the alarming worldwide rise of resistance to critically important ꞵ-lactam antibiotics^5,56,57^. Genes encoding ESBLs and carbapenamases have been increasingly reported in wild species, which is worrying since they are commonly located in mobile genetic elements that allow their rapid and efficient dissemination through horizontal gene transfer^14,58–60^.

While still describing a low prevalence, there have been a few reports of carbapenem-resistant bacteria found in wildlife^12,14,23,60,61^. Fortunately, in the present study, none of the isolates showed resistance to the tested carbapenems, meropenem and imipenem. This result greatly contrasts with the study published by Fernandes et al.^23^, in which 55% of isolates from two-toed and three-toed sloths sampled in a rehabilitation facility in Costa Rica showed resistance to meropenem. While this discrepancy should be further analysed, it is a relieving result that stays in line with the current literature and agrees with previous studies on isolates from tapirs and dogs from Costa Rica^50,62,63^.

All *E. coli* isolates under study were also susceptible to amikacin and gentamicin, the two aminoglycosides tested, supporting previous reports of a generally low prevalence of resistance to these compounds among isolates from wild animal species^23,39,43,44,64,65^.

According to the definition established by Magiorakos et al.^37^ for *Enterobacteriaceae*, almost half of the *E. coli* isolates (48%) analysed in the present study can be classified as MDR.

At least one MDR isolate was obtained in samples from 14 of the 17 analysed animal species. Although no statistically significant association was found between animal feeding habits and a MDR profile, it was observed that the samples of carnivorous animals presented a higher proportion of multidrug-resistant isolates (83.3%), particularly when compared to the samples from herbivores (40%), but also when considering the ones from omnivores (58.3%) and myrmecovores (58.3%). The difference in sampling size between these four groups is an obvious limitation for the ability to accurately associate the multidrug-resistance profile of the isolates to the feeding habits of the sampled animals. However, these results are in accordance with the ones reported in previous studies, in which AMR and MDR levels were significantly higher in carnivores and omnivores when compared to herbivores^64,66–68^.

In this study, a statistically significant association was found between the isolates’ MAR Index and the implementation of antimicrobial treatment, with isolates from treated animals having a higher MAR Index mean value (0.206) when compared to the isolates from nontreated ones (0.092). Antibiotic use is generally pointed as one of the main drivers of AMR. However, studies about antimicrobial resistance in wildlife have not been able to consistently corroborate a causal relationship between the emergence of AMR and the implementation of antimicrobial treatment. Some studies have actually stated that, when it comes to rehabilitated wildlife, the total duration of the rehabilitation or hospitalization periods seem to have a bigger impact on the development of antimicrobial resistance than the use of antibiotics per se^40,45,69^. This was not observed in the present study, as the relationship between total time in rehabilitation and both the MAR Index and multidrug-resistance profiles of the isolates was not significantly different.

While in this study the total rehabilitation time might not have been a decisive factor for the development of AMR, the rehabilitation process in general seemed to be, as both the MAR Index and MDR were significantly related to the status of the sampled animal, defined as “admitted” or “rehabilitated”. Isolates from rehabilitated animals had a higher MAR Index mean value (0.180) and a more frequent MDR profile (70%). This was an expected result, as multiple studies have reported that wild animals in captivity, rehabilitation or with an overall closer proximity to humans tend to have a higher prevalence of antibiotic-resistant bacteria^40,43,45,70–75^. Furthermore, isolates obtained from rehabilitated animals presented a higher percentage of resistance to critically important antimicrobials, such as third-generation cephalosporins and fluoroquinolones, which has also been previously observed^40,43,45^.

Some of the intrinsic characteristics of wildlife rehabilitation may contribute to the development and dissemination of AMR. Firstly, it inevitably requires some degree of direct contact between humans and animals, as the process involves capture, examination and administration of treatments, amongst other procedures that require handling by a human technician^40,71,75^. Secondly, as the main causes of admission are usually injuries and sickness, the vast majority of the admitted animals eventually have to be treated with antimicrobials, as they often present clinical signs compatible with bacterial infections. However, the implementation of antibiotic treatment in wildlife rehabilitation often poses a challenge to the veterinary practitioner, starting from the fact that the choice of active compounds and dosages are often extrapolated from what is known about companion animals or livestock, which might not always be accurate^76,77^, but also from the fact that there is often a lack of resources available to consistently perform bacterial cultures and antibiograms before implementing a treatment plan^78–80^.

This highlights the importance of prioritizing responsible antibiotic use and implementing effective biosecurity measures during the wildlife rehabilitation process, as well as the necessity to further understand exactly how these resistances might be developing and what could be the consequences for the natural ecosystems once these animals are released^40,43,71,74^. This is particularly important since, due to the current events threatening biodiversity, in the future, wildlife rehabilitation might play an increasingly prominent role in the conservation of wild animal species^11^.

However, the rehabilitation process is most probably not the only source of AMR bacteria in the analysed animal species, since high levels of antimicrobial resistance were also observed within the isolates collected from admitted animals. As these animals were sampled before the rehabilitation process or implementation of treatment, the observed resistances were likely acquired in their natural habitats.

The emergence of AMR in wildlife has been linked to the level of anthropization of the habitat in question^11,16,43^. Costa Rica has managed to maintain its forest area preserved, covering more than 50% of the country’s surface^81,82^. However, there are currently two important economic sectors in Costa Rica that deeply depend on these natural ecosystems and are highly present in the two study locations: agriculture and ecotourism.

Agriculture and livestock activities in Costa Rica should be highlighted as a potentially important source of antimicrobial resistance for wildlife. Penicillins and oxytetracycline, two of the compounds associated with the highest resistance levels observed in this study, are the most common antimicrobials applied to livestock in Costa Rica^83–85^. Multiple authors have reported malpractices in antibiotic use by Costa Rican farmers, applied to both livestock and crops^83–88^, as well as the presence of unregulated antimicrobial residues in animal feed samples^89–91^. Moreover, the national census have reported that the vast majority of farmers do not treat their wastewaters and use composting to treat their manure^92,93^, practices that have been repeatedly suggested as important sources of resistant bacteria and antimicrobial residues for the environment and wildlife^15,42,58,94,95^.

On the other hand, despite being less damaging for the environment, as well as an important source of income for conservation efforts, ecotourism in Costa Rica has been growing in a way that threatens its sustainability^81^. As this activity mainly takes place within natural ecosystems, its overdevelopment inevitably affects wild species and might increase the possibilities of AMR transmission.

International travel by itself has been shown to prompt the global spread of resistant microorganisms, potentially contributing to the emergence of novel resistant strains within Costa Rica^96,97^. Ecotourism development, as well as the increasing number of tourists visiting national parks and conservation areas, not only increases the proximity between humans and wild animals^98–100^, but can also contribute to environmental pollution and an overload of the waste management and sanitation services, which in turn can also be responsible for the dissemination of resistant strains^81,101,102^. Therefore, it is essential to maintain the sustainability of this economic sector, by balancing the increasing number of tourists with the implementation of strict regulamentation and proper management of this activity^81,101^.

These are some examples of possible sources of AMR acquisition by wildlife, considering the geographical and economic characteristics of the locations where the sampling for this study took place. However, the ecology of AMR within wildlife is extremely complex and still poorly understood, as it involves both direct and indirect interactions between various sectors, some of which might be getting overlooked.

Regarding the virulence factors studied, it was observed that most of the isolates were able to produce α-haemolysin (96%) and biofilms (76%). In this case, biofilm production is particularly important, as it is believed to increase antimicrobial resistance, and is often related to therapeutic failures and the establishment of chronic infections^103,104^. In the present study, there was no statistically significant association between the ability to produce biofilms and the MDR profile or MAR Index of the isolates, which while supporting the results from some previous studies^104–106^, is not in accordance with others^27,103,107^. During the development of resistance, mutations can lead to a possible inhibition of DNA topoisomerases and a reduction in the degree of DNA supercoiling, resulting in the reduction of some virulence gene expression or even a partial or total loss of mobile pathogenicity islands (PAIs). Therefore, the absence of a significant difference between isolates’ biofilm-forming ability and their MDR profile or MAR Index, suggests a disturbance in virulence gene expression or of the PAIs islands^108,109^.

It was possible to observe a significant negative correlation between the MAR and Virulence indexes, meaning that when the MAR Index was higher, the Virulence Index tended to be lower and vice-versa. Multiple studies have observed a negative relationship between these two factors^110–113^. Some state that there might be a trade-off between virulence and resistance to maintain bacterial fitness, as expressing both is metabolically taxing on the microorganisms^104,112,114^. Others have hypothesized that some of the mutations necessary for antimicrobial resistance might interfere with the expression of some virulence factors^111,112,115^. Nevertheless, it is increasingly accepted that, in the future, pathogens will evolve to be both highly resistant and highly virulent. This highlights the importance of further understanding the ecology of both virulence and resistance determinants in all “One Health” settings.

While the present study allowed us to draw some important conclusions regarding the antimicrobial resistance and virulence determinants currently present in Costa Rican wildlife, including both captive and free-ranging animals, its limitations must be considered in order to improve future studies. The conventional bacteriological methods applied, such as the use of culture-based approaches, biochemical tests and phenotypical evaluations, provide relatively low sensitivity and specificity, especially when compared with the molecular methods that have been increasingly used in scientific research^13,23,71,116^. The option to only characterize one isolate per sample disregards the within-host diversity of bacterial colonies^67^. Lastly, this was a cross-sectional study, meaning it is not possible to establish actual causality, only association between variables at a certain point in time. In future studies, it would be interesting to perform a longitudinal study aiming to determine if wildlife rehabilitation does in fact contribute to the appearance of antimicrobial resistance in wild species^43,117^.

This study supports previous studies that reported that wild animal species may be carriers of important resistance and virulence determinants with unknown impact for public health^11,14,18,23^. Characterizing the current status of antimicrobial resistance in wildlife is an important first step to gain awareness about its dissemination within a given ecosystem. However, although AMR in wild animals might pose a threat to public health, the resistant strains they present seem to mainly be a consequence of human action. Therefore, greater effort should be made to further investigate the actual sources of the resistance determinants affecting wildlife, since any control measure implemented to deal with this problem will only be effective if the source of resistance is addressed.

Due to a growing human population and increased habitat fragmentation, there will be a rising proximity between humans and wild animal species. Therefore, it is essential to include wildlife in future studies and surveillance programs in order to fully understand its role in the ecology of antimicrobial resistance and hopefully develop cooperative strategies to address this urgent global challenge.

## Conclusions

This study reinforced the hypothesis that wild animals might be important carriers of resistance and virulence determinants that can threaten public health. Therefore, it is fundamental to understand how these factors are emerging and disseminating within wildlife and its environment, as multiple sources can be currently pointed out and the consequences are still unknown. Future studies are encouraged to include longitudinal sampling and molecular techniques in order to identify the genetic diversity and evolution of AMR strains within wild species, as well as to allocate specific sources of resistance determinants.

As AMR is a phenomenon that involves complex interactions between humans, animals and the environment they share, it is essential to address it with a holistic and interdisciplinary approach that recognizes and understands the role of each compartment and their interactions. Therefore, including wildlife in the effort to understand the AMR problematic is crucial for the successful implementation of a “One Health” approach which, in turn, is the only hope to develop effective strategies to prevent and control this potential global health crisis.

### Supplementary Information

Supplementary Information.

## Data Availability

The dataset used during the current study are available from the corresponding author on reasonable request.
