# Supplementary material for: Resistant Escherichia coli isolated from wild mammals from two rescue and rehabilitation centers in Costa Rica: characterization and public health relevance
Source: Sci Rep. 2024 Apr 5;14:8039. doi: 10.1038/s41598-024-57812-6 (PMC10997758; doi:10.1038/s41598-024-57812-6)
Supplement: Supplementary file 1 — Supplementary Information. [file 41598_2024_57812_MOESM1_ESM.pdf]

Resistant *Escherichia coli* Isolated from Wild Mammals from Two Rescue and Rehabilitation Centers in Costa Rica: Characterization and Public Health Relevance

Scientific Reports

Rita Fernandes [1,2], Raquel Abreu [1,2], Isa Serrano [1, 2], Roger Such [3], Encarnación Garcia-Vila [3], Sandy Quirós [4], Eva Cunha [1,2], Luís Tavares [1,2] and Manuela Oliveira [1,2,5,*]

[1] CIISA - Centro de Investigação Interdisciplinar em Sanidade Animal, Faculdade de Medicina Veterinária, Universidade de Lisboa, Av. da Universidade Técnica, 1300-477 Lisboa, Portugal.
[2] AL4AnimalS - Associate Laboratory for Animal and Veterinary Sciences (AL4AnimalS), Portugal
[3] Jaguar Rescue Center, Límon, Costa Rica
[4] Alturas Wildlife Sanctuary, Puntarenas, Costa Rica
[5] cE3c - Centre for Ecology, Evolution and Environmental Changes & CHANGE - Global Change and Sustainability Institute, Faculdade de Ciências, Universidade de Lisboa
***** Correspondence: moliveira@fmv.ulisboa.pt; Tel.: +351-213602052

Supplementary Materials
Table S1 - Data of the sampled animals

Sample ID	Sampling Location	Sampling Type	Sampling Date	Scientific Name	Common Name	Sex	Age	Cause of Admission	Outcome	Antibiotic Treatment*	Time in Rehabilitation	Feeding Behaviour
AE1	AWS	Admission	14/12/2021	*Saimiri oerstedii*	Central American Squirrel Monkey	M	Adult	Injury	Euthanasia	EFT	-	O
AE2	AWS	Admission	14/12/2021	*Bradypus variegatus*	Brown-Throated Three-Toed Sloth	M	Baby	Sickness	-	-	-	H
AE3	AWS	Admission	14/12/2021	*Procyon cancrivorous*	Crab-Eating Racoon	M	Adult	Traffic Accident	Euthanasia	-	-	O
AE4	AWS	Admission	16/12/2021	*Didelphis marsupialis*	Common Opossum	M	Adult	Traffic Accident	Euthanasia	-	-	O
AE5	AWS	Admission	16/12/2021	*Dasyprocta punctata*	Central American Agouti	F	Adult	Injury	Death	-	-	H
AE6	AWS	Admission	17/12/2021	*Leopardus weidii*	Margay	M	Baby	Confiscation	-	-	-	C
AE7	AWS	Admission	16/12/2021	*Bradypus variegatus*	Brown-Throated Three-Toed Sloth	F	Adult	Sickness	Euthanasia	ENR	-	H
AE8	AWS	Admission	16/12/2021	*Cebus imitator*	White-Faced Capuchin Monkey	F	Adult	Sickness	Euthanasia	-	-	O
AE9	AWS	Admission	21/12/2021	*Choloepus hoffmanni*	Hoffmann's Two-Toed Sloth	F	Adult	Sickness	Euthanasia	-	-	H
AE10	AWS	Admission	24/12/2021	*Tamandua mexicana*	Northern Tamandua	F	Adult	Injury	-	ENR	-	MV
AE11	AWS	Admission	31/12/2021	*Leopardus pardalis*	Ocelot	M	Adult	Injury	-	-	-	C
AE12	AWS	Admission	02/01/2022	*Tamandua mexicana*	Northern Tamandua	M	Juvenile	Injury	Death	-	-	MV
AE13	AWS	Admission	08/01/2022	*Bradypus variegatus*	Brown-Throated Three-Toed Sloth	M	Adult	Sickness	Euthanasia	-	-	H
AE14	AWS	Admission	09/01/2022	*Procyon cancrivorous*	Crab-Eating Racoon	M	Adult	Traffic Accident	-	-	-	O
AE15	AWS	Admission	17/01/2022	*Leopardus weidii*	Margay	M	Juvenile	Traffic Accident	Release	-	-	C
AE16	AWS	Admission	29/01/2022	*Didelphis marsupialis*	Common Opossum	F	Adult	Injury	Death	-	-	O
AE17	AWS	Admission	31/01/2022	*Dasyprocta punctata*	Central American Agouti	M	Adult	Traffic Accident	Death	-	-	H
AE18	AWS	Admission	02/02/2022	*Tamandua mexicana*	Northern Tamandua	M	Baby	Orphan	-	-	-	MV

Legend: Alturas Wildlife Sanctuary (AWS), Jaguar Rescue Center (JRC), Male (M), Female (F), days (d); Antibiotic treatment applied before the moment of sampling (*); Ceftiofur (EFT); Enrofloxacin (ENR), Amoxicillin/Clavulanate (AMC), Trimethoprim/Sulfamethoxazole (SXT), Herbivore (H), Carnivore (C), Omnivore (O), Myrmecovore (MV)

Sample ID	Sampling Location	Sampling Type	Sampling Date	Scientific Name	Common Name	Sex	Age	Cause of Admission	Outcome	Antibiotic Treatment*	Time in Rehabilitation	Feeding Behaviour
AE19	AWS	Admission	11/02/2022	*Alouatta palliata*	Mantled Howler Monkey	M	Baby	Injury	Euthanasia	-	-	H
AS1	AWS	Rehabilitation	20/12/2022	*Philander opossum*	Common Gray Four-Eyed Opossum	M	Adult	Orphan	Release	-	-	O
AS2	AWS	Rehabilitation	09/01/2022	*Choloepus hoffmanni*	Hoffmann's Two-Toed Sloth	M	Adult	Translocation	Release	-	-	H
AS3	AWS	Rehabilitation	14/01/2022	*Cuniculus paca*	Paca	F	Adult	Injury	Release	-	< 1 d	H
AS4	AWS	Rehabilitation	14/01/2022	*Cuniculus paca*	Paca	F	Adult	Confiscation	Release	-	< 1 d	H
AS5	AWS	Rehabilitation	14/01/2022	*Cuniculus paca*	Paca	F	Adult	Confiscation	Release	-	38 d	H
AS6	AWS	Rehabilitation	14/01/2022	*Cuniculus paca*	Paca	F	Adult	Confiscation	Release	-	38 d	H
AS7	AWS	Rehabilitation	14/01/2022	*Cuniculus paca*	Paca	M	Adult	Confiscation	Release	-	38 d	H
AS8	AWS	Rehabilitation	14/01/2022	*Leopardus pardalis*	Ocelot	M	Adult	Injury	Release	AMC	38 d	C
AS10	AWS	Rehabilitation	04/02/2022	*Tamandua mexicana*	Northern Tamandua	M	Juvenile	Orphan	Release	AMC	20 d	MV
AS11	AWS	Rehabilitation	16/02/2022	*Puma yagouaroundi*	Jaguarundi	F	Juvenile	Orphan	Euthanasia	-	270 d	C
JE1	JRC	Admission	02/03/2022	*Bradypus variegatus*	Brown-Throated Three-Toed Sloth	M	Adult	Injury	Death	ENR	184 d	H
JE2	JRC	Admission	04/03/2022	*Choloepus hoffmanni*	Hoffmann's Two-Toed Sloth	F	Juvenile	Translocation	Release	-	-	H
JE3	JRC	Admission	05/03/2022	*Alouatta palliata*	Mantled Howler Monkey	M	Adult	Injury	Euthanasia	-	-	H
JE4	JRC	Admission	06/03/2022	*Bradypus variegatus*	Brown-Throated Three-Toed Sloth	F	Adult	Translocation	Release	-	-	H
JE5	JRC	Admission	10/03/2022	*Choloepus hoffmanni*	Hoffmann's Two-Toed Sloth	F	Baby	Orphan	Death	-	-	H
JE6	JRC	Admission	13/03/2022	*Bradypus variegatus*	Brown-Throated Three-Toed Sloth	M	Adult	Electrocution	Death	-	-	H
JE7	JRC	Admission	14/03/2022	*Alouatta palliata*	Mantled Howler Monkey	F	Adult	Traffic Accident	Release	-	-	H
JE8	JRC	Admission	18/03/2022	*Alouatta palliata*	Mantled Howler Monkey	F	Adult	Electrocution	Death	-	-	H
JE9	JRC	Admission	22/03/2022	*Dasyprocta punctata*	Central American Agouti	M	Juvenile	Sickness	Death	-	-	H
JE10	JRC	Admission	23/03/2022	*Dasyprocta punctata*	Central American Agouti	M	Adult	Injury	Death	AMC	-	H

Legend: Alturas Wildlife Sanctuary (AWS), Jaguar Rescue Center (JRC), Male (M), Female (F), days (d); Antibiotic treatment applied before the moment of sampling (*); Ceftiofur (EFT); Enrofloxacin (ENR), Amoxicillin/Clavulanate (AMC), Trimethoprim/Sulfamethoxazole (SXT), Herbivore (H), Carnivore (C), Omnivore (O), Myrmecovore (MV)

Sample ID	Sampling Location	Sampling Type	Sampling Date	Scientific Name	Common Name	Sex	Age	Cause of Admission	Outcome	Antibiotic Treatment*	Time in Rehabilitation	Feeding Behaviour
JE11	JRC	Admission	23/03/2022	*Alouatta palliata*	Mantled Howler Monkey	F	Baby	Sickness	Death	-	-	H
JE12	JRC	Admission	23/03/2022	*Choloepus hoffmanni*	Hoffmann's Two-Toed Sloth	M	Juvenile	Injury	Death	-	-	H
JE13	JRC	Admission	25/03/2022	*Choloepus hoffmanni*	Hoffmann's Two-Toed Sloth	F	Adult	Injury	Death	-	-	H
JE14	JRC	Admission	29/03/2022	*Dasyprocta punctata*	Central American Agouti	F	Adult	Sickness	Death	-	-	H
JE15	JRC	Admission	29/03/2022	*Choloepus hoffmanni*	Hoffmann's Two-Toed Sloth	M	Adult	Injury	-	-	-	H
JE16	JRC	Admission	31/03/2022	*Choloepus hoffmanni*	Hoffmann's Two-Toed Sloth	F	Adult	Injury	Release	-	-	H
JE17	JRC	Admission	01/04/2022	*Alouatta palliata*	Mantled Howler Monkey	F	Juvenile	Sickness	Death	-	-	H
JE18	JRC	Admission	05/04/2022	*Choloepus hoffmanni*	Hoffmann's Two-Toed Sloth	M	Baby	Orphan	Death	-	-	H
JE19	JRC	Admission	05/04/2022	*Choloepus hoffmanni*	Hoffmann's Two-Toed Sloth	F	Adult	Death	Death	-	-	H
JE20	JRC	Admission	08/04/2022	*Choloepus hoffmanni*	Hoffmann's Two-Toed Sloth	F	Adult	Electrocution	Death	-	-	H
JE21	JRC	Admission	10/04/2022	*Choloepus hoffmanni*	Hoffmann's Two-Toed Sloth	F	Juvenile	Sickness	Death	-	-	H
JE22	JRC	Admission	10/04/2022	*Tamandua mexicana*	Northern Tamandua	M	Adult	Traffic Accident	Death	-	-	MV
JE23	JRC	Admission	12/04/2022	*Alouatta palliata*	Mantled Howler Monkey	F	Adult	Injury	Death	-	-	H
JE24	JRC	Admission	12/04/2022	*Didelphis marsupialis*	Common Opossum	F	Adult	Translocation	Release	-	-	O
JE25	JRC	Admission	12/04/2022	*Procyon lotor*	Northern Racoon	M	Adult	Sickness	-	-	-	O
JE26	JRC	Admission	15/04/2022	*Artibeus obscurus*	Dark Fruit-Eating Bat	M	Juvenile	Injury	Euthanasia	-	-	H
JE27	JRC	Admission	16/04/2022	*Caluromys derbianus*	Central American Woolly Opossum	M	Adult	Electrocution	Death	-	-	O
JE28	JRC	Admission	16/04/2022	*Choloepus hoffmanni*	Hoffmann's Two-Toed Sloth	F	Baby	Sickness	-	-	-	H
JE29	JRC	Admission	16/04/2022	*Bradypus variegatus*	Brown-Throated Three-Toed Sloth	M	Adult	Translocation	Release	-	-	H
JE30	JRC	Admission	21/04/2022	*Alouatta palliata*	Mantled Howler Monkey	M	Adult	Sickness	Release	-	-	H
JE31	JRC	Admission	24/04/2022	*Bradypus variegatus*	Brown-Throated Three-Toed Sloth	F	Adult	Sickness	-	-	-	H

Legend: Alturas Wildlife Sanctuary (AWS), Jaguar Rescue Center (JRC), Male (M), Female (F), days (d); Antibiotic treatment applied before the moment of sampling (*); Ceftiofur (EFT); Enrofloxacin (ENR), Amoxicillin/Clavulanate (AMC), Trimethoprim/Sulfamethoxazole (SXT), Herbivore (H), Carnivore (C), Omnivore (O), Myrmecovore (MV)

Sample ID	Sampling Location	Sampling Type	Sampling Date	Scientific Name	Common Name	Sex	Age	Cause of Admission	Outcome	Antibiotic Treatment*	Time in Rehabilitation	Feeding Behaviour
JE32	JRC	Admission	25/04/2022	*Eira barbara*	Tayra	M	Adult	Traffic Accident	Death	-	-	O
JE33	JRC	Admission	27/04/2022	*Didelphis marsupialis*	Common Opossum	M	Adult	Injury	-	-	-	O
JE34	JRC	Admission	30/04/2022	*Bradypus variegatus*	Brown-Throated Three-Toed Sloth	F	Juvenile	Sickness	-	-	-	H
JE35	JRC	Admission	30/04/2022	*Choloepus hoffmanni*	Hoffmann's Two-Toed Sloth	M	Juvenile	Sickness	-	-	-	H
JE36	JRC	Admission	30/04/2022	*Dasyprocta punctata*	Central American Agouti	M	Adult	Injury	Euthanasia	-	-	H
JS1	JRC	Rehabilitation	07/03/2022	*Choloepus hoffmanni*	Hoffmann's Two-Toed Sloth	M	Adult	Orphan	Release	STX	1125 d	H
JS2	JRC	Rehabilitation	07/03/2022	*Choloepus hoffmanni*	Hoffmann's Two-Toed Sloth	F	Adult	Orphan	Release	-	1125 d	H
JS3	JRC	Rehabilitation	12/03/2022	*Caluromys derbianus*	Central American Woolly Opossum	F	Juvenile	Orphan	Release	-	70 d	O
JS4	JRC	Rehabilitation	12/03/2022	*Caluromys derbianus*	Central American Woolly Opossum	M	Juvenile	Orphan	Release	-	70 d	O
JS5	JRC	Rehabilitation	29/03/2022	*Philander opossum*	Common Gray Four-Eyed Opossum	F	Juvenile	Orphan	Release	-	60 d	O
JS6	JRC	Rehabilitation	02/04/2022	*Alouatta palliata*	Mantled Howler Monkey	M	Adult	Orphan	Release	-	455 d	H
JS7	JRC	Rehabilitation	02/04/2022	*Alouatta palliata*	Mantled Howler Monkey	F	Adult	Orphan	Release	STX	940 d	H
JS8	JRC	Rehabilitation	02/04/2022	*Alouatta palliata*	Mantled Howler Monkey	F	Adult	Orphan	Release	STX	515 d	H
JS9	JRC	Rehabilitation	11/04/2022	*Choloepus hoffmanni*	Hoffmann's Two-Toed Sloth	M	Adult	Injury	Release	-	11 d	H
JS10	JRC	Rehabilitation	16/04/2022	*Coendou mexicanus*	Mexican Hairy Dwarf Porcupine	M	Adult	Injury	Release	ENR	63 d	H
JS11	JRC	Rehabilitation	18/04/2022	*Procyon lotor*	Northern Racoon	M	Adult	Orphan	Release	Metronidazol	247 d	O
JS12	JRC	Rehabilitation	18/04/2022	*Procyon lotor*	Northern Racoon	M	Adult	Orphan	Release	Metronidazol	247 d	O

Legend: Alturas Wildlife Sanctuary (AWS), Jaguar Rescue Center (JRC), Male (M), Female (F), days (d); Antibiotic treatment applied before the moment of sampling (*); Ceftiofur (EFT); Enrofloxacin (ENR), Amoxicillin/Clavulanate (AMC), Trimethoprim/Sulfamethoxazole (SXT), Herbivore (H), Carnivore (C), Omnivore (O), Myrmecovore (MV)

Table S2 - Results of the isolates' resistance and virulence profiles

ID	Sampling Location	Species	Feeding Behaviour	MAR. Index	Resistance Profile		V. Index	Virulence Profile					
					R	I		PT	DNA	GEL	LEC	HEM	BF
AE3.C	AWS	*Procyon cancrivorous*	O	0.067	EFT	AMP, OT	0,500	+	-	-	-	α	+
AE9.D	AWS	*Choloepus hoffmanni*	H	0.200	AMP, CL, OT	-	0,333	+	-	-	-	α	-
AE10.A	AWS	*Tamandua mexicana*	M	0.133	AZM, OT	CIP, ENR	0,500	+	-	-	-	α	+
AE13.D	AWS	*Bradypus variegatus*	H	0.000	-	-	0,500	+	-	-	-	α	+
AE14.B	AWS	*Procyon cancrivorous*	O	0.000	-	AMP	0,333	+	-	-	-	α	-
AE15.C	AWS	*Leopardus weidii*	CV	0.200	AMP, CAZ, CL	-	0,167	-	-	-	-	-	+
AE19.A	AWS	*Alouatta palliata*	H	0.133	CL, ENR	OT	0,333	-	-	-	-	α	+
AS2.A	AWS	*Choloepus hoffmanni*	H	0.067	CIP	AMP	0,500	+	-	-	-	α	+
AS8.D	AWS	*Leopardus pardalis*	CV	0.533	AMP, C, CAZ, CIP, CL, EFT, ENR, OT	AMC	0,333	-	-	-	-	α	+
AS10.E	AWS	*Tamandua mexicana*	M	0.067	CL	AMP	0,333	+	-	-	-	α	-
AS11.D	AWS	*Puma yagouaroundii*	CV	0.133	AMP, CL	OT	0,167	-	-	-	-	α	-
JE1.D	JRC	*Bradypus variegatus*	H	0.067	CL	AMC, OT	0,333	-	-	-	-	α	+
JE2.C	JRC	*Choloepus hoffmanni*	H	0.133	AMP, OT	-	0,500	+	-	-	-	α	+
JE3.B	JRC	*Alouatta palliata*	H	0.067	CL	AMP	0,333	-	-	-	-	α	+
JE3.D	JRC	*Alouatta palliata*	H	0.067	CL	AMP	0,167	-	-	-	-	α	-
JE4.B	JRC	*Bradypus variegatus*	H	0.067	CL	AMP	0,333	-	-	-	-	α	+
JE5.D	JRC	*Choloepus hoffmanni*	H	0.067	CL	AMP, OT	0,500	+	-	-	-	α	+
JE6.D	JRC	*Bradypus variegatus*	H	0.000	-	AMP	0,333	-	-	-	-	α	+

Legend: Alturas Wildlife Sanctuary (AWS), Jaguar Rescue Center (JRC), Positive (+), Negative (-), Herbivore (H), Carnivore (CV), Omnivore (O), Myrmecovore (M) Amoxicillin/Clavulanic Acid (AMC), Cephalexin (CL), Ceftazidime (CAZ), Ceftiofur (EFT), Ciprofloxacin (CIP), Enrofloxacin (ENR), Azithromycin (AZM), Ampicillin (AMP), Chloramphenicol (C), Trimethoprim/Sulfamethoxazole (SXT), Oxytetracycline (OT)

ID	Sampling Location	Species	Feeding Behaviour	MAR. Index	Resistance Profile		V. Index	Virulence Profile					
					R	I		PT	DNA	GEL	LEC	HEM	BF
JE7.B	JRC	*Alouatta palliata*	H	0.067	CL	AMP	0,333	-	-	-	-	α	+
JE8.C	JRC	*Alouatta palliata*	H	0.000	-	-	0,333	+	-	-	-	α	-
JE9.A	JRC	*Dasyprocta punctata*	H	0.000	-	OT	0,333	-	-	-	-	α	+
JE10.A	JRC	*Dasyprocta punctata*	H	0.133	AMP, AZM	-	0,333	-	-	-	-	α	+
JE10.D	JRC	*Dasyprocta punctata*	H	0.133	AMP, CL	-	0,500	+	-	-	-	α	+
JE11.A	JRC	*Alouatta palliata*	H	0.067	CL	-	0,500	+	-	-	-	α	+
JE12.B	JRC	*Choloepus hoffmanni*	H	0.067	CL	AMP	0,500	+	-	-	-	α	+
JE13.D	JRC	*Choloepus hoffmanni*	H	0.000	-	AMP	0,500	+	-	-	-	α	+
JE15.D	JRC	*Choloepus hoffmanni*	H	0.000	-	-	0,000	-	-	-	-	-	-
JE16.A	JRC	*Choloepus hoffmanni*	H	0.133	AMP, CL	OT	0,333	-	-	-	-	α	+
JE17.C	JRC	*Alouatta palliata*	H	0.067	AMP	-	0,333	-	-	-	-	α	+
JE18.B	JRC	*Choloepus hoffmanni*	H	0.000	-	-	0,333	-	-	-	-	α	+
JE20.B	JRC	*Choloepus hoffmanni*	H	0.133	CAZ, CL	AMP	0,333	-	-	-	-	α	+
JE21.D	JRC	*Choloepus hoffmanni*	H	0.000	-	AMP	0,333	-	-	-	-	α	+
JE22.C	JRC	*Tamandua mexicana*	M	0.133	AMP, CL	OT	0,333	-	-	-	-	α	+
JE23.B	JRC	*Alouatta palliata*	H	0.067	CL	OT	0,333	-	-	-	-	α	+
JE24.A	JRC	*Didelphis marsupialis*	O	0.133	AMP, OT	-	0,167	-	-	-	-	α	-
JE28.B	JRC	*Choloepus hoffmanni*	H	0.067	AZM	AMP, OT	0,167	-	-	-	-	α	-
JE31.D	JRC	*Bradypus variegatus*	H	0.000	-	-	0,500	-	+	-	-	α	+
JE29.B	JRC	*Bradypus variegatus*	H	0.133	AMP, AZM	OT	0,167	-	-	-	-	α	-

Legend: Alturas Wildlife Sanctuary (AWS), Jaguar Rescue Center (JRC), Positive (+), Negative (-), Herbivore (H), Carnivore (CV), Omnivore (O), Myrmecovore (M) Amoxicillin/Clavulanic Acid (AMC), Cephalexin (CL), Ceftazidime (CAZ), Ceftiofur (EFT), Ciprofloxacin (CIP), Enrofloxacin (ENR), Azithromycin (AZM), Ampicillin (AMP), Chloramphenicol (C), Trimethoprim/Sulfamethoxazole (SXT), Oxytetracycline (OT)

ID	Sampling Location	Species	Feeding Behaviour	MAR. Index	Resistance Profile		V. Index	Virulence Profile					
					R	I		PT	DNA	GEL	LEC	HEM	BF
JE30.A	JRC	*Alouatta palliata*	H	0.00	AMP, AZM, C, CIP, ENR, OT	-	0,333	-	-	-	-	α	+
JE30.C	JRC	*Alouatta palliata*	H	0.33	AMP, CL	OT	0,167	-	-	-	-	α	-
JE32.A	JRC	*Eira barbara*	O	0.067	CL	AMP	0,333	-	-	-	-	α	+
JE32.D	JRC	*Eira barbara*	O	0.067	CL	AMP, OT	0,333	-	-	-	-	α	+
JE34.A	JRC	*Bradypus variegatus*	H	0.067	CL	AMP	0,500	-	+	-	-	α	+
JE35.B	JRC	*Choloepus hoffmanni*	H	0.000	-	AMP	0,333	-	+	-	-	-	+
JE36.B	JRC	*Dasyprocta punctata*	H	0.067	CL	AMP	0,333	-	-	-	-	α	+
JS1.A	JRC	*Choloepus hoffmanni*	H	0.267	AZM, CIP, ENR, OT	-	0,167	-	-	-	-	α	-
JS3.A	JRC	*Caluromys derbianus*	H	0.067	AMP	-	0,500	+	-	-	-	α	+
JS5.B	JRC	*Philander opossum*	O	0.133	AMP, CL	OT, AMC	0,833	+	+	+	-	α	+
JS5.C	JRC	*Philander opossum*	O	0.200	AMP, CL, OT	AMC	0,333	-	-	-	-	α	+
JS7.D	JRC	*Alouatta palliata*	H	0.067	OT	AMP	0,500	+	-	-	-	α	+
JS9.B	JRC	*Choloepus hoffmanni*	H	0.200	CL, ENR, OT	AMP, CIP	0,333	-	-	-	-	α	+
JS9.D	JRC	*Choloepus hoffmanni*	H	0.067	OT	-	0,500	+	-	-	-	α	+
JS10.B	JRC	*Coendou mexicanus*	H	0.600	AMP, C, CAZ, CIP, CL, EFT, ENR, OT, STX	-	0,333	-	-	-	-	α	+
JS10.D	JRC	*Coendou mexicanus*	H	0.133	AMP, CL	AMC, OT	0,333	-	-	-	-	α	+
JS11.D	JRC	*Procyon lotor*	O	0.133	AMP, CL	AMC, OT	0,333	-	-	-	-	α	+
JS12.C	JRC	*Procyon lotor*	O	0.200	AMC, CL, CAZ	-	0,500	-	+	-	-	α	+
AE8.B	AWS	*Sebus imitator*	O	0.133	AMP, CL	AMC, OT	0,333	-	-	-	-	α	+
AE8.C	AWS	*Sebus imitator*	O	0.133	AMP, CL	AMC, OT	0,333	-	-	-	-	α	+

Legend: Alturas Wildlife Sanctuary (AWS), Jaguar Rescue Center (JRC), Positive (+), Negative (-), Herbivore (H), Carnivore (CV), Omnivore (O), Myrmecovore (M) Amoxicillin/Clavulanic Acid (AMC), Cephalexin (CL), Ceftazidime (CAZ), Ceftiofur (EFT), Ciprofloxacin (CIP), Enrofloxacin (ENR), Azithromycin (AZM), Ampicillin (AMP), Chloramphenicol (C), Trimethoprim/Sulfamethoxazole (SXT), Oxytetracycline (OT)

ID	Sampling Location	Species	Feeding Behaviour	MAR. Index	Resistance Profile		V. Index	Virulence Profile					
					R	I		PT	DNA	GEL	LEC	HEM	BF
AE11.C	AWS	*Leopardus pardalis*	CV	0.133	AMP, CL	AMC, OT	0,333	-	-	-	-	α	+
AE16.C	AWS	*Didelphis marsupialis*	O	0.000	-	OT	0,500	+	-	-	-	α	+
AE16.D	AWS	*Didelphis marsupialis*	O	0.067	OT	-	0,333	-	-	-	-	α	+
AE18.B	AWS	*Tamandua mexicana*	M	0.133	AMP, CL	CAZ, OT	0,333	-	-	-	-	α	+
AS4.A	AWS	*Cuniculus paca*	H	0.133	AMP, CL	OT	0,333	+	-	-	-	α	-
AS5.A	AWS	*Cuniculus paca*	H	0.200	AMP, CL, OT	AMC	0,167	-	-	-	-	α	-
AS5.D	AWS	*Cuniculus paca*	H	0.200	AMP, CL, OT	-	0,167	-	-	-	-	α	-
AS6.A	AWS	*Cuniculus paca*	H	0.133	AMP, CL	OT	0,333	+	-	-	-	α	-
AS7.C	AWS	*Cuniculus paca*	H	0.067	AMP	-	0,333	-	-	-	-	α	+

Legend: Alturas Wildlife Sanctuary (AWS), Jaguar Rescue Center (JRC), Positive (+), Negative (-), Herbivore (H), Carnivore (CV), Omnivore (O), Myrmecovore (M) Amoxicillin/Clavulanic Acid (AMC), Cephalexin (CL), Ceftazidime (CAZ), Ceftiofur (EFT), Ciprofloxacin (CIP), Enrofloxacin (ENR), Azithromycin (AZM), Ampicillin (AMP), Chloramphenicol (C), Trimethoprim/Sulfamethoxazole (SXT), Oxytetracycline (OT)

Table S3 – Statistical Results

Pearson's Chi-Square Test

**Variables tested**		**Chi-square value**	***p*-value**
Location of Sampling (AWS/JRC)	Biofilm Production (+/-)	1.4756	0.2245
Multidrug-Resistance (Y/N)	Biofilm Production (Y/N)	2.8079	0.0938
	Antibiotherapy (Y/N)	1.7733	0.1830

Legend: Alturas Wildlife Sanctuary (AWS), Jaguar Rescue Center (JRC), Yes (Y), No (N), Positive (+), Negative (-)

Fisher's Exact Test

**Variables tested**		***p*-value**
Animal's Feeding Habits (H/O/C/M)	E. coli result (+/-)	0.1406
	Multidrug Resistance (Y/N)	0.1254
Biofilm Production (+/-)	DNAse Production (+/-)	0.3272
	Haemolysis Production (+/-)	0.5653
	Protease Production (+/-)	0.5512
	Gelatinase Production (+/-)	0.9981

Legend: Yes (Y), No (N), Positive (+), Negative (-), Herbivore (H), Omnivore (O), Carnivore (C), Myrmecovore (M)

Spearman Correlation Test

**Variables tested**		**Spearman Correlation Coefficient**	***p*-value**
MAR Index	V. Index	-0.2594	0.0339*

Legend: Multiple Antimicrobial Resistance Index (MAR Index), Virulence Index (V. Index), statistically significant value (*)

Mann-Whitney U Test

Variables Tested		U value	*p*-value
MAR Index	Location of Sampling (AWS/JRC)	604	0.1325
	Animal Status (A/R)	236	0.0008*
	Sex (M/F)	541	0.8294
	Antibiotherapy (Y/N)	180.5	0.0101*
	Biofilm Production (+/-)	474	0.3171
V. Index	Location of Sampling (AWS/JRC)	459.5	0.6009
	Animal Status (A/R)	462	0.9084
	Sex (M/F)	641	0.2455
	Antibiotherapy (Y/N)	286	0.4259

Legend: Multiple Antimicrobial Resistance Index (MAR Index), Virulence Index (V. Index), Alturas Wildlife Sanctuary (AWS), Jaguar Rescue Center (JRC), Yes (Y), No (N), Positive (+), Negative (-), Male (M), Female (F), Admitted (A), Rehabilitated (R), statistically significant value (*)

Kruskal-Wallis Test

Variables tested		H value	*p*-value
MAR Index	Species	24.965	0.0700
	Age	1.1496	0.5628
	Cause of Admission	8.5626	0.1997
	Time in Rehabilitation	0.2629	0.8768
	Feeding Behaviour	3.1696	0.3662
V. Index	Species	14.273	0.5784
	Age	1.0410	0.5942
	Cause of Admission	5.7978	0.4462
	Time in Rehabilitation	0.19998	0.9048
	Feeding Behaviour	4.5973	0.2038
